# Polymorphisms in estrogen receptors predict the risk of male infertility: a meta-analysis

**DOI:** 10.1186/1477-7827-12-79

**Published:** 2014-08-16

**Authors:** Tian-Fu Li, Qiu-Yue Wu, Cui Zhang, Wei-Wei Li, Na Li, Ying-Xia Cui, Xiao-Jun Li, Xin-Yi Xia

**Affiliations:** Department of Reproduction and Genetics, Institute of Laboratory Medicine, Jinling Hospital, Nanjing University School of Medicine, Nanjing, 210002 PR China

**Keywords:** Male infertility, Polymorphisms, Estrogen receptors

## Abstract

**Background:**

Estrogen receptors play an important role in mediating estrogen action on target tissues, and the estrogen is relevant to male infertility. Single nucleotide polymorphisms (SNPs) in estrogen receptors may be associated with the risk of male infertility. A variety of case control studies have been published evaluating this association. However, the accumulated studies have shown inconsistent conclusions.

**Methods:**

To further determine the potential association between the four common SNPs (rs2234693, rs9340799, rs1256049 and rs4986938) in estrogen receptors gene and male infertility, this meta-analysis was performed according to the 10 published case control studies. The odds ratio (OR) and 95% confidence interval (CI) were used to evaluate the strength of the associations.

**Results:**

It was revealed that the sub-group analysis by the ethnicity, for the rs2234693, a significant association in the comparison of CC vs. TT (OR = 0.61, 95% CI: 0.40-0.93), CT vs. TT (OR = 0.67, 95% CI: 0.49-0.93) and CC + CT vs. TT (OR = 0.66, 95% CI: 0.49-0.89) in the Asian population with male infertility. For rs9340799 polymorphism, increased risks were observed for the comparison of AA vs. GG (OR = 1.75, 95% CI: 1.15-2.68) and AA vs. GA + GG (OR = 1.38, 95% CI: 1.02-1.88). For rs1256049 polymorphism, the comparison of the GA vs. GG (OR = 1.52, 95% CI: 1.00-2.31) and AA + GA vs. GG (OR = 1.74, 95% CI: 1.03-2.94), also increased risks present in Asian and Caucasian population, respectively.

**Conclusions:**

The rs2234693C allele was associated with the decreased risk for male infertility; however, the rs9340799AA genotype and the rs1256049GA genotype were associated with an increased risk for male infertility.

**Electronic supplementary material:**

The online version of this article (doi:10.1186/1477-7827-12-79) contains supplementary material, which is available to authorized users.

## Background

Male infertility is an important cause of couple’s inability to bear children in 20% to 25% of total cases and the etiology of nearly half of the cases remains idiopathic [1, 2]. Approximately 15% of male infertile cases, genetic factors, including chromosomal aberrations and single gene mutations, may result in spermatogenic failure and sperm dysfunction [3, 4]. The traditional view of estradiol as the ‘female’ hormone and of testosterone as the ‘male’ hormone has been challenged due to the increased interest in elucidating the role of estrogen in males [5]. Estrogens are produced in the male reproductive system by Sertoli cells, Leydig cells, and germ cells [6, 7]. In addition, studies revealed that estrogens reduce testosterone production from Leydig cells and reduce Sertoli cell numbers in adult when they are given during development [8, 9]. The estrogens can also disrupt fetal Leydig cell development, inhibit apoptosis of human postmeiotic germ cells, and increase spermatogonial number per testis [8–12]. The physical functions of estrogens were involved in the estrogen receptors (ERs). Moreover, ERs are members of the nuclear receptor (NR) superfamily that mediates the pleiotropic effects of estrogen in a diverse range of developmental and physiological processes, playing an important role in mediating estrogen action on target tissues [13, 14].

ERs have been identified to be two subtypes of ERα and ERβ. ERα is a 595-amino acid protein [15] encoded by the *ERs1* gene on chromosome 6q25, and ERβ is a 530-amino acid protein [16] which encoded by the *ERs2* gene on chromosome 14q22-24 [17]. Genetic screening for the ERα gene locus has revealed several polymorphic sites [18], and two polymorphisms located in ERα intron 1(T/C transition, rs2234693) and in 50 bp downstream of the former one (G/A transition, rs9340799) have been widely concerned. In addition, the ERβ genes have been described with two silent G/A polymorphisms (rs1256049 and rs4986938) [19]. To date, epidemiological studies have been carried out to evaluate the association between ER polymorphisms and male infertility. However, the results remain inconsistent (Table 1) [5, 7, 19–26]. In order to get a more precise estimation of the association between polymorphisms in ERs and risk of male infertility, this meta-analysis was performed based on ten eligible previously published studies.

**Table 1:** **Summary of published studies included**

Author	Year	Race	Source of control	Method	Polymorphism sites	Characteristics of study patients	Case/control counts	HWE (Control)
Meng [19]	2013	Asian	PB	PCR-RFLP	rs2234693, rs9340799, rs1256049, rs4986938	Age: 25–38 years (mean age 32.1 ± 5.2 years). Exclusion criteria: abnormal karyotypes, deletions of the Y chromosome, orchitis, varicocele, cryptorchidism, congenital bilateral absence of the vas deferens, hypogonadotropic hypogonadism, and iatrogenic infertility.	TT:83/82, CT:96/126, CC:25/44; AA:151/148, AG:42/89,GG:11/15; GG:103/127, AG:91/102, AA:10/23; GG:155/193, AG:41/48, AA:8/11	0.712, 0.793, 0.699, 0.001
Zalata [5]	2013	Caucasian	PB	PCR-RFLP	rs2234693, rs9340799	Inclusion criteria: same ethnic origin (Caucasians). Exclusion criteria: varicocele, hormonal therapy, hypogonadism, smoking, Y chromosome deletions and karyotype abnormalities. The ages of were not shown in the article.	TT:33/14, CT:32/27, CC:16/19; AA:28/8, AG:36/32, GG: 17/20	0.468, 0.389
Ogata [20]	2012	Asian	PB	PCR-RFLP	rs1256049	Age: 32–52 years (median 41.0 years). Inclusion criteria: no extragenital anomalies, seminal tract obstruction, varicocele, Y chromosomal microdeletion, or retrograde ejaculation; normal karyotypes.	GG:68/64, AG:49/45, AA:8/10	0.604
Bianco [7]	2011	Caucasian	PB	TaqMan assays	rs2234693, rs9340799, rs1256049, rs4986938	Age: 36.1 ± 6.5 years. Exclusion criteria: chromosome anomalies, azoospermia factor (AZF) microdeletions, smoking, alcoholism, occupation, varicocele, and cryptorchidism.	TT:30/37, CT:93/111, CC:64/68; AA:80/100, AG:79/88, GG:20/28; GG:172/201, AG:15/15, AA:0/0; GG:43/28, AG:60/103, AA:84/85	0.468, 0.221, 0.597, 0.712
Safarinejad [21]	2010	Asian	PB	PCR-RFLP	rs2234693, rs9340799, rs1256049, rs4986938	Age: 31.6 ± 4.8 years (range 25–40 years). Inclusion criterion: two years with no reason for their infertility. Exclusion criteria: varicocele or testicular torsion, urinary tract infections, endocrinopathy, karyotype anomalies, Y-chromosome microdeletions, use of drugs, leukocytospermia, a BMI of 30 kg/m2 or greater.	TT:49/33, CT:70/86, CC:45/45; AA:62/41, AG:77/95, GG:25/28; GG:142/152, AG:21/8, AA:1/4; GG:65/80, AG:82/63, AA:17/21	0.486, 0.034, 0.000, 0.132
Lazaros [22]	2010	Caucasian	PB	PCR-RFLP	rs2234693, rs9340799, rs1256049, rs4986938	Age: 33.2 ± 67.5 years. Exclusion criteria: hypogonadotropic hypogonadism, obstructive syndromes of the seminal tract, microdeletions of the Y chromosome, karyotypic abnormalities.	TT:6/20, CT:14/40, CC:9/25; AA:5/13, AG:13/43, GG:11/29; GG:26/80, AG:3/5, AA:0/0; GG:7/17, AG:12/36, AA:10/32	0.609, 0.652, 0.779, 0.246
Khattri [23]	2007	Asian	PB	PCR-RFLP	rs1256049	Age: 23.24 ± 2.06 years. Exclusion criteria: obstruction, endocrinological defect, injuries, karyotypic abnormality, Y-chromosome microdeletions.	GG:397/231, AG:46/21, AA:0/0	0.490
Omrani [24]	2005	Asian	PB	PCR-RFLP	rs1256049, rs4986938	Exclusion criteria: genetic causes of infertility, such as Klinefelter syndrome or Ychromosome microdeletions. The ages of patients were no shown in the article.	GG:103/194, AG:17/9, AA:0/1; GG:51/86, AG:57/88,AA:12/30	0.023, 0.339
Aschim [25]	2005	Caucasian	PB	PCR-RFLP	rs1256049, rs4986938	Exclusion criteria: Klinefelter syndrome or Y-chromosome microdeletions, a history of cryptorchidism were excluded. The ages of patients were no shown in the article.	GG:92/177, AG:14/8, AA:0/1; GG:11/82, AG:48/79, AA:47/25	0.015, 0.394
Kukuvitis [26]	2002	Caucasian	PB	PCR-RFLP	rs2234693, rs9340799	Exclusion criteria: any known aetiologies (varicocele, infections of accessory glands, cryptorchidism, homozygous form of β-thalassemia). The ages of patients were no shown in the article.	TT:38/18, CT:38/25, CC:33/21; AA:30/10, AG:45/28, GG:34/26	0.083, 0.594

PB, Population Based; PCR-RFLP, Polymerase Chain Reaction–restriction Fragment Length Polymorphism; HWE, Hardy–Weinberg equilibrium; BMI, body mass index.

## Methods

### Identification and eligibility of studies

To identify all articles that examined the association of ERs polymorphisms with male infertility, a comprehensive systematic bibliographic search through the medical databases PUBMED, attempting to cover all medical papers published between 1950 and 2013, using the following keywords and subject terms: “male infertility”, “polymorphism” and “estrogen receptors” or “ERs”. The synonyms of polymorphism (rs2234693, rs9340799, rs1256049, and rs4986938) were also used as the keywords in the search. The studies were excluded if they were not English language papers or human subject. References in retrieved articles were screened in which case reports, meta-analyses and review articles were excluded. In addition, studies were identified by a manual search of the references lists of reviews and retrieved studies. All the studies were included if they met the following criteria: (I) about the rs2234693, rs9340799, rs1256049, and rs4986938 polymorphisms and male infertility, (II) from a case control study, (III) genotype frequencies could be derived. The reasons for exclusion of articles were listed in the Additional file 1: Text S1.

### Data extraction

Two authors (Tian-Fu Li and Qiu-Yue Wu) extracted all data independently that met the inclusion criteria and reached the consensus for any controversy. The main characteristics of the enrolled studies were listed in the Table 1, including: (I) the first author’s last name, (II) year of publication, (III) ethnicity, (IV) source of control groups (population- or hospital-based controls), (V) genotyping methods, (VI) the polymorphism sites, (VII) characteristics of studies, (VIII) Case/Control counts, (IX) Hardy–Weinberg equilibrium in the controls. Data were extracted separately for each ethnic groups categorized as Caucasian and Asian. However, no African was identified in this study.

### Statistical analysis

The risk of male infertility associated with the four polymorphisms of the ERs gene was estimated for each study by odds ratio (OR), together with its 95% confidence interval (CI), respectively. The four polymorphisms were evaluated for the associations with male infertility susceptibility based on four genetic models. To contrast, the wild-type homozygote (WW), we first estimated the risk of the rare allele homozygote (RR) and heterozygous (WR) genotypes on infertility, then evaluated the risk of infertility under a dominant model (RR + WR vs. WW). In addition, recessive model associations were also estimated (RR vs. WR + WW). Moreover, stratified analyses were also performed by ethnicity (Asian and Caucasian). The statistical significance of the pooled OR was determined with the Z-test and a P-value of <0.05 was considered significant. Heterogeneity across the studies was evaluated by Chi-square test based on Q test [27] and was considered significant if *P* <0.05.A fixed-effect model using the Mantel–Haenszel method and a random-effects model using the DerSimonian and Laird method were used to pool the results [28]. In addition, the fixed-effect model was used as well when there was no heterogeneity across results of the studies, or the random-effect model. Moreover, a sensitivity analysis, by which a single study in the meta-analysis was deleted each time to determine the influence of the individual data set to the overall pooled OR, was performed to assess the stability of the results. To test the publication bias, Funnel plots and Egger’s linear regression test were applied [29]. Hardy–Weinberg equilibrium in the controls of each study was calculated using a web-based program [30]. All statistical tests for this meta-analysis were performed with STATA version 10.0 (Stata Corporation College Station, TX, USA).

## Results

### Characteristics of studies

**Figure 1:**
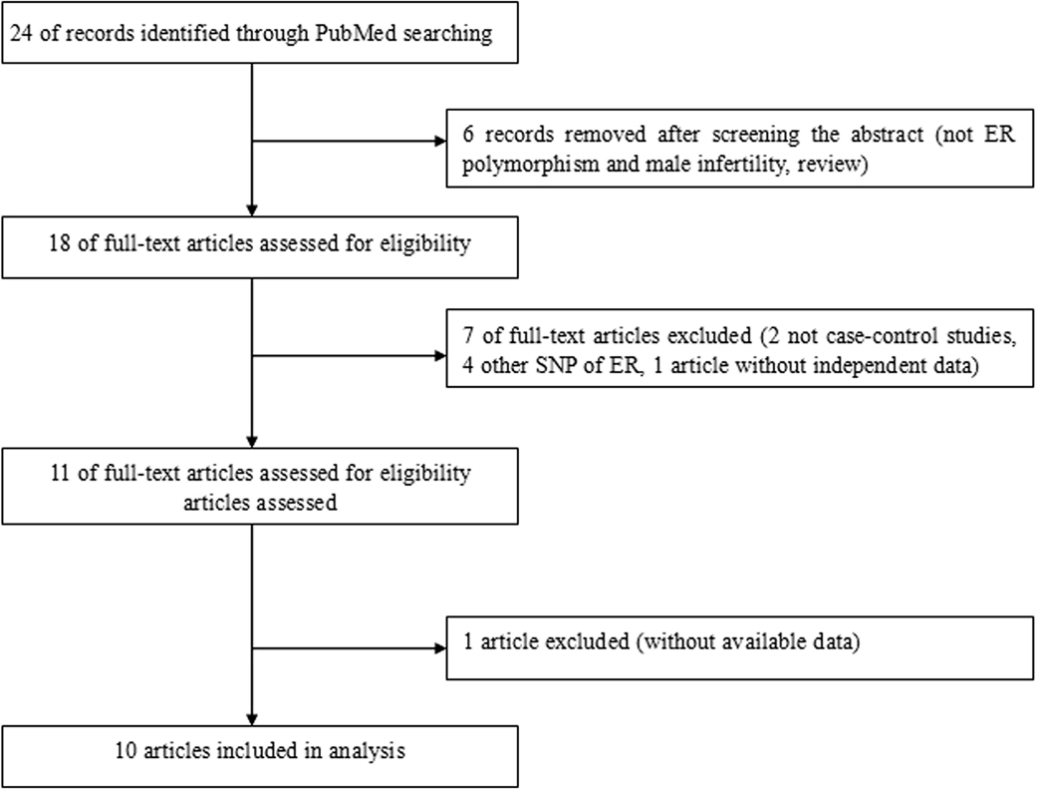
**Flow chart of studies identified with inclusion and exclusion criteria.**

A total of 10 eligible case control studies with the publication dates ranged from 2002 to 2013 met the prespecified inclusion criteria (shown in the Figure 1), including five studies of Asian population [19–21, 23, 24] and five studies of Caucasian population [5, 7, 22, 25, 26]. To determine the SNPs, two different genotyping methods such as PCR-RFLP [5, 19–26] and TaqMan assays [7] were applied. All subjects were received comprehensive andrological examination, and the patients were divided into three types: oligozoospermia (sperm count <20 × 10^6^/mL), azoospermia and oligoasthenoteratozoospermic (OAT). The studies’ exclusion criteria and inclusion criteria were listed in the Table 1. In addition, the sources of controls in these studies were mainly population-based. The distribution of genotypes in the controls of all studies was consistent with Hardy–Weinberg equilibrium except for the study [19] in rs4986938, the study [21] in rs9340799 and rs1256049, study [24] in rs 1256049 and study [25] in rs1256049, which were tested in the sensitivity analyses.

### Quantitative synthesis

**Figure 2:**
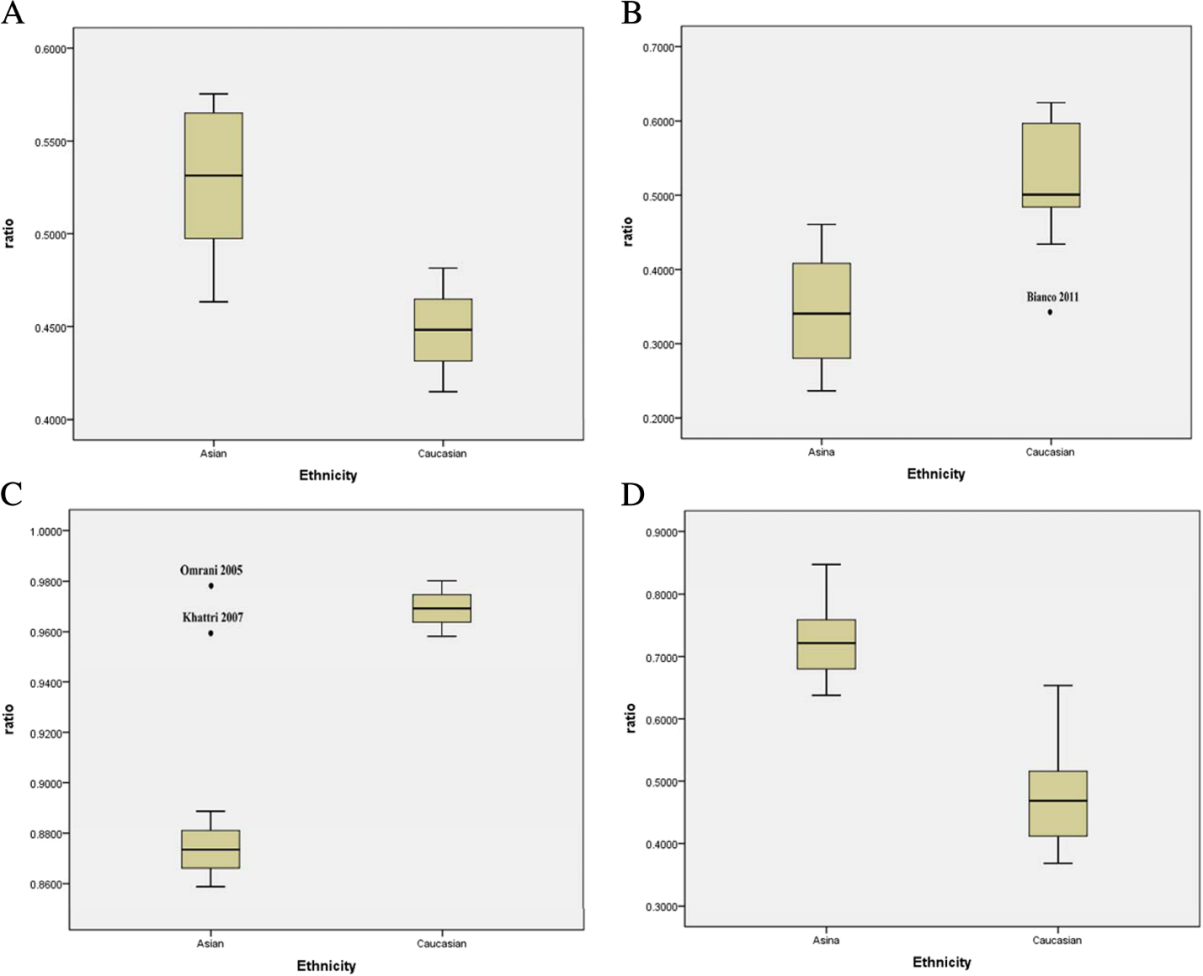
**The allel frequencies of the four polymorphisms in the controls may vary by ethnicity. (A)** rs2234693, **(B)** rs9340799, **(C)** rs1256049, **(D)** rs4986938. Star or dot denotes outliers.

Wide variation of four polymorphisms allele frequencies across different ethnicities was observed. For rs2234693, the frequency of T allele was 53.13% (95% CI: 49.74-56.52) in the Asian controls, which was higher than that in Caucasian controls 44.82% (95% CI: 41.48-48.16) as shown in Figure 2A. For rs9340799, the frequency of G allele in the Asian controls (32.45%, 95% CI: 29.27-35.63) was lower than that in Caucasian controls (46.71%, 95% CI: 43.36-50.06) as shown in Figure 2B. In Figure 2C, we could find that the frequency of G allele for the rs1256049 in the Asian controls (87.34%, 95% CI: 85.88-88.81) was lower than which in Caucasian controls (96.92%, 95% CI: 95.82-98.02). In contrast, the frequency of G allele in Caucasian controls (48.46%, 95% CI: 45.32-51.60) was lower than that in Asian group (73.39%, 95% CI: 70.93-75.85) for the rs4986938 in Figure 2D.

For rs2234693 polymorphism, significant differences were observed for the comparison of CC vs. TT, CT vs. TT and CC + CT vs. TT. Sub-group analysis by the ethnicity revealed a significant association in the comparison of CC vs. TT (OR = 0.61, 95% CI: 0.40-0.93, *P*_heterogeneity_ = 0.670), CT vs. TT (OR = 0.67, 95% CI: 0.49-0.93, *P*_heterogeneity_ = 0.358), CC + CT vs. TT (OR = 0.66, 95% CI: 0.49-0.89, *P*_heterogeneity_ = 0.593) and C alleles vs. T alleles (OR = 0.78, 95% CI: 0.64-0.96, *P*_heterogeneity_ = 0.681) in the Asian population, as summarized in Table 2.

**Table 2:** **Stratification analyses of genetic susceptibility of rs2234693 polymorphism to male infertility**

Category	Cases/controls	CC vs. TT			CT vs. TT			CC + CT vs. TT			CC vs. CT + TT			C allele vs. T allele		
		OR(95% CI)	***P*** ^a^	***I*** ^2^	OR(95% CI)	***P*** ^a^	***I*** ^2^	OR(95% CI)	***P*** ^a^	***I*** ^2^	OR(95% CI)	***P*** ^a^	***I*** ^2^	OR(95% CI)	***P*** ^a^	***I*** ^2^
Total	774/841	**0.72(0.54-0.96)**	0.282	20.1	**0.74(0.58-0.94)**	0.533	0	**0.73(0.58-0.91)**	0.368	7.5	0.90(0.71-1.13)	0.465	0	0.84(0.71-1.01)	0.190	32.8
***RACE***																
Asian	368/416	**0.61(0.40-0.93)**	0.670	0	**0.67(0.49-0.93)**	0.358	0	**0.66(0.49-0.89)**	0.593	0	0.83(0.58-1.18)	0.257	22.1	**0.78(0.64-0.96)**	0.681	0
Caucasian	406/425	0.83(0.46-1.23)	0.175	39.5	0.83(0.57-1.19)	0.460	0	0.81(0.58-1.14)	0.232	30	0.95(0.70-1.29)	0.395	0	0.87(0.64-1.18)	0.103	51.5

*I* ^2^: 0–25, no heterogeneity; 25–50, modest.

^a^ *P* value of Q-test for heterogeneity test.

Bold numbers mean statistically significant results.

For rs9340799 polymorphism, increased risks were observed for the comparison of AA vs. GG and AA vs. GA + GG. Sub-group analysis by ethnicity revealed increased risks (AA vs. GG: OR = 1.75, 95% CI: 1.15-2.68, *P*_heterogeneity_ = 0.174; AA vs. GA + GG: OR = 1.38, 95% CI: 1.02-1.88, *P*_heterogeneity_ = 0.062) in the Caucasian population, also for the AA vs. GA + GG and A alleles vs. G alleles, a significant association was observed in Asian population (OR = 1.93, 95% CI: 1.42-2.62, *P*_heterogeneity_ = 0.768; OR = 1.49, 95% CI: 1.18-1.87, *P*_heterogeneity_ = 0.375) as summarized in Table 3.

**Table 3:** **Stratification analyses of genetic susceptibility of rs9340799 polymorphism to male infertility**

Category	Cases/controls	AA vs. GG			GA vs. GG			AA + GA vs. GG			AA vs. GA + GG			A allele vs. G allele		
		OR(95% CI)	***P*** ^a^	***I*** ^2^	OR(95% CI)	***P*** ^a^	***I*** ^2^	OR(95% CI)	***P*** ^a^	***I*** ^2^	OR(95% CI)	***P*** ^a^	***I*** ^2^	OR(95% CI)	***P*** ^a^	***I*** ^2^
Total	774/841	**1.67(1.21-2.32)**	0.392	3.9	1.03(0.76-1.39)	0.764	0	1.27(0.96-1.68)	0.796	0	**1.63(1.32-2.03)**	0.077	49.7	**1.39(1.13-1.68)**	0.172	35.3
***RACE***																
Asian	368/416	1.56(0.93-2.62)	0.714	0	0.81(0.49-1.34)	0.524	0	1.13(0.71-1.82)	0.952	0	**1.93(1.42-2.62)**	0.768	0	**1.49(1.18-1.86)**	0.375	0
Caucasian	406/425	**1.75(1.15-2.68)**	0.174	39.7	1.17(0.81-1.71)	0.847	0	1.35(0.95-1.92)	0.569	0	**1.38(1.02-1.88)**	0.062	59.2	1.39(0.97-1.81)	0.109	50.5

^a^ *P* value of Q-test for heterogeneity test.

*I* ^2^: 0–25, no heterogeneity; 25–50, modest heterogeneity; 50, high heterogeneity.

Bold numbers mean statistically significant results.

For rs1256049 polymorphism, significant differences were observed for the comparison of GA vs. GG, AA + GA vs. GG and AA vs. GA + GG. For the comparison of the GA vs. GG, AA + GA vs. GG, increased risks present in Asian and Caucasian population, respectively (GA vs. GG: OR = 1.52, 95% CI: 1.00-2.31, *P*_heterogeneity_ = 0.038; AA + GA vs. GG: OR = 1.74, 95% CI: 1.03-2.94, *P*_heterogeneity_ = 0.275). All data were concluded in the Table 4. In contrast, a decreased risk was also observed for the comparison AA vs. GA + GG (OR = 0.55, 95% CI: 0.31-0.97, *P*_heterogeneity_ = 0.818) in Asian population. For the rs4986938, there was no significant association observed in all comparisons (data were not shown).

**Table 4:** **Stratification analyses of genetic susceptibility of rs1256049 polymorphism to male infertility**

Category	Cases/Controls	AA vs. GG			GA vs. GG			AA + GA vs. GG			AA vs. GA + GG			A allele vs. G allele		
		OR(95% CI)	***P*** ^a^	***I*** ^2^	OR(95% CI)	***P*** ^a^	***I*** ^2^	OR(95% CI)	***P*** ^a^	***I*** ^2^	OR(95% CI)	***P*** ^a^	***I*** ^2^	OR(95% CI)	***P*** ^a^	***I*** ^2^
Total	1378/1478	0.57(0.32-1.01)	0.940	0	**1.59(1.12-2.25)** ^**b**^	**0.047**	50.9	**1.30(1.05-1.61)**	0.075	45.7	**0.55(0.32-0.96)**	0.920	0	1.29(0.97-1.72)	0.068	46.9
***RACE***																
Asian	1056/991	0.57(0.32-1.01)	0.853	0	**1.52(1.00-2.31)** ^**b**^	**0.038**	60.6	1.23(0.98-1.56)	0.064	55	**0.55(0.31-0.97)**	0.818	0	1.19(0.86-1.65)	0.067	54.4
Caucasian	322/487	0.64(0.03-15.86)	-	-	1.87(0.92-3.80)	0.208	36.2	**1.74(1.03-2.94)**	0.275	22.6	0.58(0.02-14.38)	-	-	1.66(0.99-2.77)	0.372	0

^a^ *P* value of Q-test for heterogeneity test.

^b^Random-effects model was used when a *P* value, 0.05 for heterogeneity test; otherwise, fixed-effects model was used.

*I* ^2^: 0–25, no heterogeneity; 25–50, modest heterogeneity; 50, high heterogeneity.

Bold numbers mean statistically significant results.

### Test of heterogeneity

**Figure 3:**
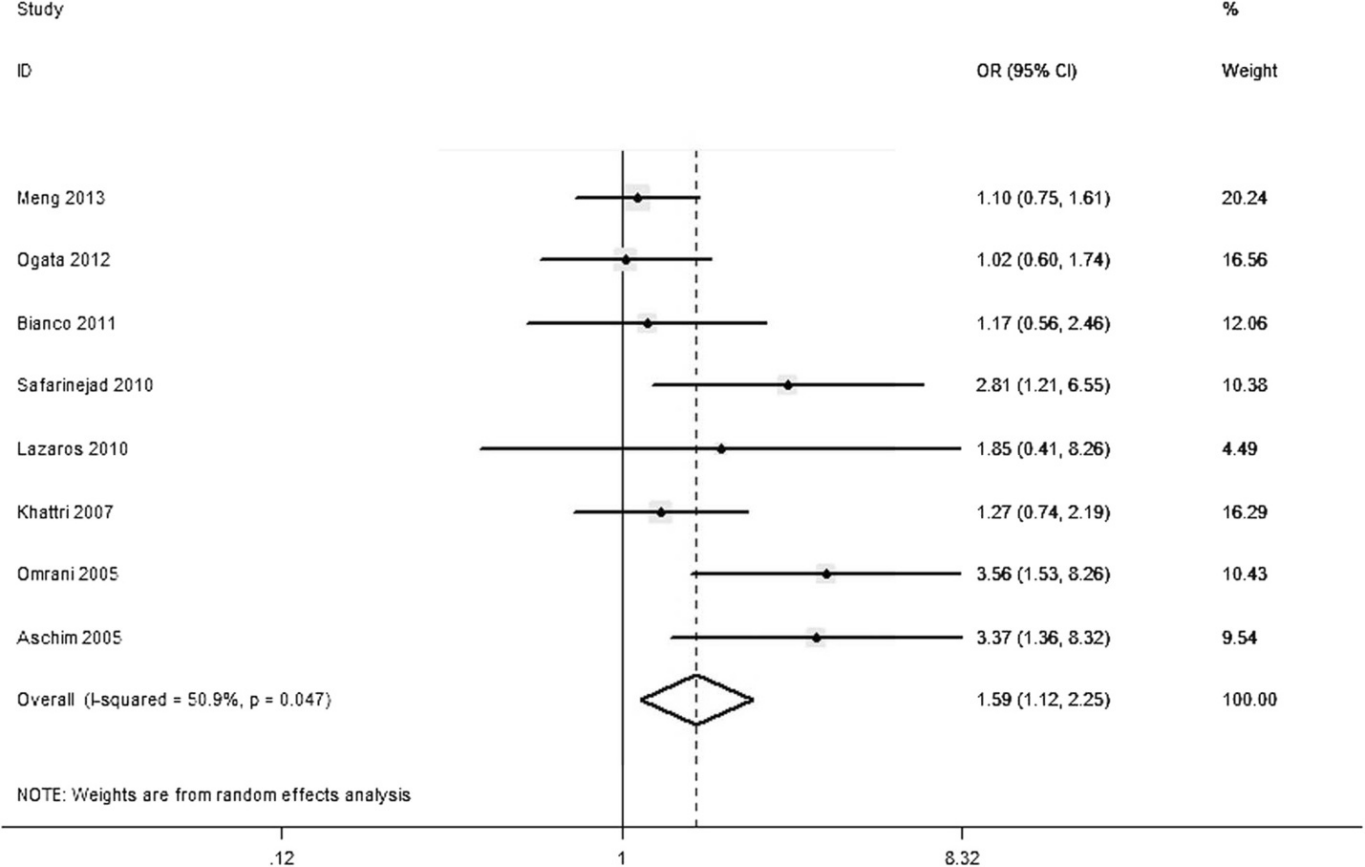
**Forest plot for the overall association between rs1256049 polymorphism and male infertility for random effects.** For GA vs. GG each study was shown by the point estimate of the OR (the size of the *square* is proportional to the weight of each study) and 95% CI for the OR (*extending lines*); the pooled OR and 95% CI were shown by *diamonds*.

Among the four polymorphisms, a significant heterogeneity was apparent among heterozygote comparison for the rs1256049 (GA vs. GG: *P*_heterogeneity_ = 0.047) (Figure 3). Two studies [7, 19] were identified to contribute to substantial heterogeneity, and it was decreased when the study was removed respectively (*P* = 0.065, *P* = 0.075). Sensitivity analysis revealed that the two independent studies [7, 23] were the main cause of heterogeneity for the rs1256049. Heterogeneity was decreased when these studies were removed (GA vs. GG: *P*_heterogeneity_ = 0.320, *I*^*2*^ = 14.7%). Although the genotype distributions in four studies did not follow Hardy–Weinberg equilibrium, the corresponding pooled ORs were not materially altered by excluding the studies.

### Publication bias

**Figure 4:**
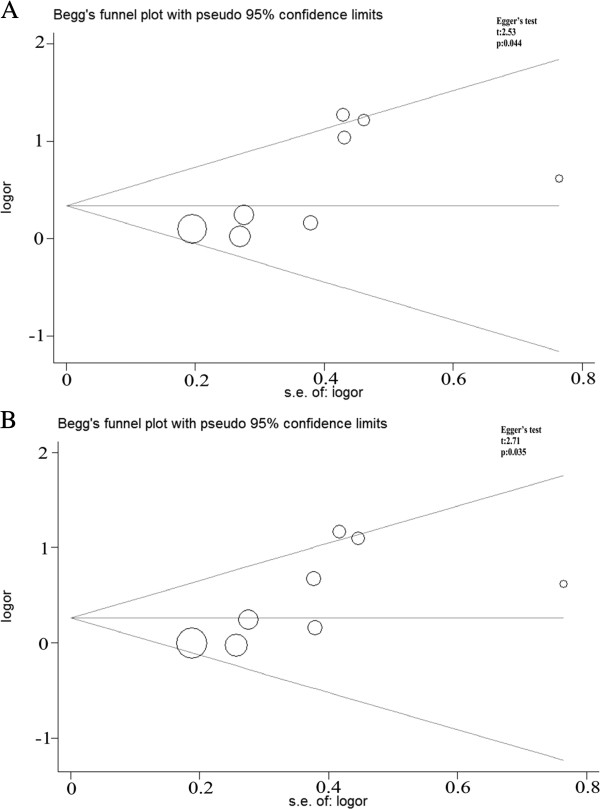
**Begg’s funnel plot of publication bias test for the rs1256049. (A)** GA vs. GG. **(B)** AA + GA vs. GG. Each point represents a separate study for the indicated association. Log (OR), natural logarithm of OR. Horizontal line means effect size.

**Figure 5:**
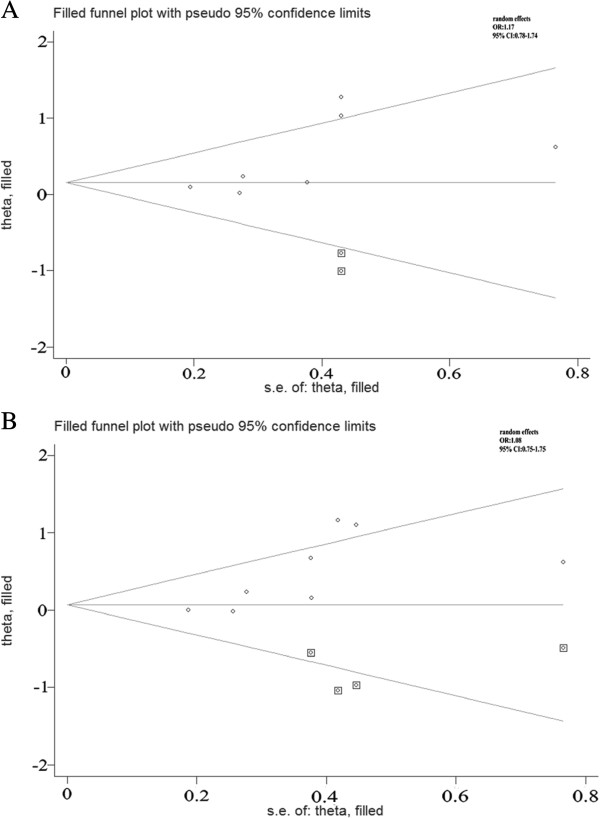
**Begg’s funnel plot of publication bias test for the rs1256049 using the trim-and-fill method. (A)** GA vs. GG. **(B)** AA + GA vs. GG. Each point represents a separate study for the indicated association. Log (OR), natural logarithm of OR. Horizontal line means effect size. The adjusted estimates obtained by using the random effects model for GA vs. GG and AA + GA vs. GG.

Begg’s funnel plot and Egger’s test were performed to assess the publication bias of the currently available literature. For the rs2234693, rs9340799 and rs4986938, the shape of the funnel plots did not reveal any evidence of obvious asymmetry in all comparison models. Then, the Egger’s test was used to provide statistical evidence for funnel plot symmetry. The results also did not show any evidence of publication bias. However, for the rs1256049, as shown in the Figure 4, the shape of the funnel plots seemed asymmetrical in the heterozygote and dominant comparisons, suggesting the presence of publication bias. Then, the Egger’s tests were adopted to provide statistical evidence of funnel plot asymmetry. As expected, the results showed obvious evidence of publication bias (*t* = 2.53, *P* = 0.044 for GA vs. GG; *t* = 2.71, *P* = 0.035 for AA + GA vs. GG). To adjust for this bias, a trim-and-fill method developed by Duval and Tweedie [31] was implemented. Trimming was based on fixed-effects model, and the adjusted estimates obtained by using the random effects model were ORs of 1.17 (0.78-1.74) for GA vs. GG and 1.08 (0.75-1.54) for AA + GA vs. GG in the Figure 5. Although Meta-analysis with or without the trim-and-fill method also ends up with same conclusions, but the ORs were not statistically significant difference. So it was indicated that the results of these studies were not statistically robust.

## Discussion

The present meta-analysis, including 1568 cases and 1602 controls from 10 case control studies, explored the association between the ERs polymorphisms and male infertility. The results indicated that rs2234693C allele was associated with decreased risk of the male infertility, particularly in the Asian population. In contrast, rs9340799AA genotype was observed as a risk factor for infertility in both Asian and Caucasian population, and rs1256049GA genotype was associated with an increased risk for developing male sterility. However, the rs4986938 polymorphism was not associated with male infertility. In addition, we tried to find the data in the available database, such as PUBMED [32], National Human Genome Research Institute GWAS Catalog [33] and GWAS Central [34], but we found no relevant genome-wide association (GWAS) study about these four polymorphisms.

Estradiol has been reported as a survival factor for germ cells [11], involving in the induction of oxidative DNA damage, and the aberrant level of estrogen may lead to impaired sperm production [35–37]. It has been shown that free radicals inhibit steriodogenesis by interfering with cholesterol transport to the mitochondria and/or the catalytic function of P450 enzymes, which leads to an increase in lipid per oxidation and decline in the antioxidant barrier [38]. Moreover, estrogens can regulate mitochondrial function by increasing nuclear respiratory factor-1 (NRF-1) expression [5]. Specifically, estradiol stimulates mitochondrial function through a genomic mechanism of ER action involving direct ERα and ERβ interaction with an oestrogen response element in the NRF-1 promoter [39]. In vivo knockdown experiments have indicated that estradiol stimulates NRF-1 transcription and consequently increases mitochondrial biogenesis through ERα activity but not through ERβ activity in MCF-7 breast cells [40]. This findings indicates that ERα polymorphisms can increase mitochondrial activity via NRF-1 transcription in human ejaculated spermatozoa, presenting them with high motility [22].

The mechanisms behind altered ERs function in subjects with polymorphisms remain unclear. The polymorphism rs1256049 located at the splice acceptor site just prior to exon 8 in ERβ [41] and may potentially affect the splicing of this exon, leading to proteins with different properties than the wild-type ERβ [42, 43]. In addition, studies have reported the polymorphism could also have a direct effect through changing the nucleotide sequence and thereby the secondary structure of the ERβ mRNA, possibly leading to changes the function of mRNA [44, 45]. It has been reported that ERα gene polymorphisms (rs2234693 and rs9340799) may modalate the effect of oestradiol on CYP19, which encodes aromatase expression, disrupting the gene causes a decline in sperm numbers and loss of male infertility [46, 47].

The precise role of estrogen receptors in male fertility status is understood. Some findings suggest that specific polymorphisms of the ERα, and ERβ genes which confer a lower sex hormone binding globulin (SHBG) and thus a stronger unbound estrogen effect, may adversely affect human spermatogenesis [48, 49]. SHBG is involved in both delivering reproductive hormones to target tissues and controlling the concentration of androgens and estrogens in the serum and tissues [50]. Pavlovich et al. [51] demonstrated that infertile men with severe oligozoospermia had significantly lower T (testosterone) and higher E2 (estradiol) concentrations than fertile control subjects, resulting in an elevated T/E2 ratio.

Identifying the source of heterogeneity is one of the most important goals of the meta-analysis. Thus, we stratified the studies only according to ethnicity (because the sources of the controls were selected through population-based, and the method used was the only one different). Stratified analysis by ethnicity revealed that there was no difference between the European population and Asian population, suggesting that different ethnicities and environmental exposures may have no influence on the susceptibility of male infertility, and more studies should be accumulated to reveal the difference. In addition, for the rs1256049, sensitivity analysis revealed that the three independent studies [7, 22, 23] were the main source of heterogeneity. Heterogeneity was decreased when these studies were removed. For these three studies, the sample size was not sufficient and the numbers of rs1256049AA genotype was both zero. These two points may be the main reason for the heterogeneity in the performed analysis. For the rs1256049, there was obvious evidence of publication bias. As the same with heterogeneity, the numbers of the cases and controls of the wild-type homozygote in these three studies [7, 22, 23] were too small to keep the results statistically robust, so it maybe the key factor for the bias. Using a proper and representative subject is very important in reducing bias in such genotype association studies.

There are still some limitations in this meta-analysis. Firstly, there were only ten literatures enrolled in this meta-analysis, the sample size was not big enough to have substantial power exploring the real association. Secondly, the detailed information (such as life-style, age, and work) could not be traced, so that our unadjusted estimates should be confirmed by further studies. In addition, an individual with a clinical disorder was not a result of the single gene that is disrupted, but that the genetic disruption was embedded within the context of that individual's entire genome and environment exposure [52]. In fact, some other genes related to fertility could also play an important role in spermatogenesis.

## Conclusions

In summary, this meta-analysis suggested that the rs2234693C allele was the protective factor for male infertility, the rs9340799AA genotype was associated with an increased risk for infertility, and the rs1256049GA genotype was also the negative factor.

## Electronic supplementary material

Additional file 1: Text S1: The reasons for exclusion of the articles which were shown in Figure 1. (DOC 77 KB)

## Abbreviations

CI: Confidence interval
E2: Estradiol
ERs: Estrogen receptors
NR: Nuclear receptor
NRF-1: Nuclear respiratory factor-1
OR: Odds ratio
PCR-RFLP: Polymerase chain reaction–restriction fragment length polymorphism
SHBG: Sex hormone binding globulin
SNPs: Single nucleotide polymorphisms
T: Testosterone
vs.: Versus.
