# Supplementary material for: Polymorphisms in estrogen receptors predict the risk of male infertility: a meta-analysis
Source: Reprod Biol Endocrinol. 2014 Aug 16;12:79. doi: 10.1186/1477-7827-12-79 (PMC4141121; doi:10.1186/1477-7827-12-79)
Supplement: Supplementary file 1 — Additional file 1: Text S1: The reasons for exclusion of the articles which were shown in Figure 1. (DOC 77 KB) [file 12958_2014_1250_MOESM1_ESM.doc]

**The reasons for exclusion of the articles which were shown in Figure 1**

1. Romerius P, Giwercman A, Moëll C, Relander T, Cavallin-Ståhl E, Wiebe T, Halldén C, Giwercman YL: **Estrogen receptor α single nucleotide polymorphism modifies the risk of azoospermia in childhood cancer survivors.** *Pharmacogenet Genomics* 2011, **21**:263-269.

(This article is not about male infertility.)

1. [Zhang QF](http://www.ncbi.nlm.nih.gov/pubmed?term=Zhang QF%5BAuthor%5D&cauthor=true&cauthor_uid=21132001), [Feng HL](http://www.ncbi.nlm.nih.gov/pubmed?term=Feng HL%5BAuthor%5D&cauthor=true&cauthor_uid=21132001), [Zhao L](http://www.ncbi.nlm.nih.gov/pubmed?term=Zhao L%5BAuthor%5D&cauthor=true&cauthor_uid=21132001), [Liu P](http://www.ncbi.nlm.nih.gov/pubmed?term=Liu P%5BAuthor%5D&cauthor=true&cauthor_uid=21132001), [Li L](http://www.ncbi.nlm.nih.gov/pubmed?term=Li L%5BAuthor%5D&cauthor=true&cauthor_uid=21132001), [Yan J](http://www.ncbi.nlm.nih.gov/pubmed?term=Yan J%5BAuthor%5D&cauthor=true&cauthor_uid=21132001), [Qiao J](http://www.ncbi.nlm.nih.gov/pubmed?term=Qiao J%5BAuthor%5D&cauthor=true&cauthor_uid=21132001): **Alteration of ERβ gene RsaI polymorphism may contribute to reduced fertilization rate and embryonic developmental competence.** *Asian J Androl* 2011, **13**:317-321.

(This article is not a case control study.)

1. Massart F, Saggese G: **Sex steroidal targets & genetic susceptibility to idiopathic cryptorchidism.** *Pediatr Endocrinol Rev* 2009, **6**:481-490.

(This article is a review.)

1. [Lee IW](http://www.ncbi.nlm.nih.gov/pubmed?term=Lee IW%5BAuthor%5D&cauthor=true&cauthor_uid=21429951), [Kuo PH](http://www.ncbi.nlm.nih.gov/pubmed?term=Kuo PH%5BAuthor%5D&cauthor=true&cauthor_uid=21429951), [Su MT](http://www.ncbi.nlm.nih.gov/pubmed?term=Su MT%5BAuthor%5D&cauthor=true&cauthor_uid=21429951), [Kuan LC](http://www.ncbi.nlm.nih.gov/pubmed?term=Kuan LC%5BAuthor%5D&cauthor=true&cauthor_uid=21429951), [Hsu CC](http://www.ncbi.nlm.nih.gov/pubmed?term=Hsu CC%5BAuthor%5D&cauthor=true&cauthor_uid=21429951), [Kuo PL](http://www.ncbi.nlm.nih.gov/pubmed?term=Kuo PL%5BAuthor%5D&cauthor=true&cauthor_uid=21429951): **Quantitative trait analysis suggests polymorphisms of estrogen-related genes regulate human sperm concentrations and motility.** *Hum Reprod* 2011, **26**:1585-1596.

(In this article, we can’t get the genotype frequencies.)

1. [Su MT](http://www.ncbi.nlm.nih.gov/pubmed?term=Su MT%5BAuthor%5D&cauthor=true&cauthor_uid=18980759)^1^, [Chen CH](http://www.ncbi.nlm.nih.gov/pubmed?term=Chen CH%5BAuthor%5D&cauthor=true&cauthor_uid=18980759), [Kuo PH](http://www.ncbi.nlm.nih.gov/pubmed?term=Kuo PH%5BAuthor%5D&cauthor=true&cauthor_uid=18980759), [Hsu CC](http://www.ncbi.nlm.nih.gov/pubmed?term=Hsu CC%5BAuthor%5D&cauthor=true&cauthor_uid=18980759), [Lee IW](http://www.ncbi.nlm.nih.gov/pubmed?term=Lee IW%5BAuthor%5D&cauthor=true&cauthor_uid=18980759), [Pan HA](http://www.ncbi.nlm.nih.gov/pubmed?term=Pan HA%5BAuthor%5D&cauthor=true&cauthor_uid=18980759), [Chen YT](http://www.ncbi.nlm.nih.gov/pubmed?term=Chen YT%5BAuthor%5D&cauthor=true&cauthor_uid=18980759), [Kuo PL](http://www.ncbi.nlm.nih.gov/pubmed?term=Kuo PL%5BAuthor%5D&cauthor=true&cauthor_uid=18980759): **Polymorphisms of estrogen-related genes jointly confer susceptibility to human spermatogenic defect.** *Fertil Steril* 2010, **93**:141-149.

(This article’s data is not independent.)

1. Krausz C, Giachini C: **Genetic risk factors in male infertility.** *Arch Androl* 2007, **53**:125-133.

(This article is a review.)

1. [Corbo RM](http://www.ncbi.nlm.nih.gov/pubmed?term=Corbo RM%5BAuthor%5D&cauthor=true&cauthor_uid=17556378), [Ulizzi L](http://www.ncbi.nlm.nih.gov/pubmed?term=Ulizzi L%5BAuthor%5D&cauthor=true&cauthor_uid=17556378), [Piombo L](http://www.ncbi.nlm.nih.gov/pubmed?term=Piombo L%5BAuthor%5D&cauthor=true&cauthor_uid=17556378), [Martinez-Labarga C](http://www.ncbi.nlm.nih.gov/pubmed?term=Martinez-Labarga C%5BAuthor%5D&cauthor=true&cauthor_uid=17556378), [De Stefano GF](http://www.ncbi.nlm.nih.gov/pubmed?term=De Stefano GF%5BAuthor%5D&cauthor=true&cauthor_uid=17556378), [Scacchi R](http://www.ncbi.nlm.nih.gov/pubmed?term=Scacchi R%5BAuthor%5D&cauthor=true&cauthor_uid=17556378): **Estrogen receptor alpha polymorphisms and fertility in populations with different reproductive patterns.** *Mol Hum Reprod* 2007, **13**:537-540.

(This article is not a case control study.)

1. [Watanabe M](http://www.ncbi.nlm.nih.gov/pubmed?term=Watanabe M%5BAuthor%5D&cauthor=true&cauthor_uid=17283037), [Yoshida R](http://www.ncbi.nlm.nih.gov/pubmed?term=Yoshida R%5BAuthor%5D&cauthor=true&cauthor_uid=17283037), [Ueoka K](http://www.ncbi.nlm.nih.gov/pubmed?term=Ueoka K%5BAuthor%5D&cauthor=true&cauthor_uid=17283037), [Aoki K](http://www.ncbi.nlm.nih.gov/pubmed?term=Aoki K%5BAuthor%5D&cauthor=true&cauthor_uid=17283037), [Sasagawa I](http://www.ncbi.nlm.nih.gov/pubmed?term=Sasagawa I%5BAuthor%5D&cauthor=true&cauthor_uid=17283037), [Hasegawa T](http://www.ncbi.nlm.nih.gov/pubmed?term=Hasegawa T%5BAuthor%5D&cauthor=true&cauthor_uid=17283037), [Sueoka K](http://www.ncbi.nlm.nih.gov/pubmed?term=Sueoka K%5BAuthor%5D&cauthor=true&cauthor_uid=17283037), [Kamatani N](http://www.ncbi.nlm.nih.gov/pubmed?term=Kamatani N%5BAuthor%5D&cauthor=true&cauthor_uid=17283037), [Yoshimura Y](http://www.ncbi.nlm.nih.gov/pubmed?term=Yoshimura Y%5BAuthor%5D&cauthor=true&cauthor_uid=17283037), [Ogata T](http://www.ncbi.nlm.nih.gov/pubmed?term=Ogata T%5BAuthor%5D&cauthor=true&cauthor_uid=17283037): **Haplotype analysis of the estrogen receptor 1 gene in male genital and reproductive abnormalities.** *Hum Reprod* 2007, **22**:1279-1284.

(The SNP in this article is a different one from the four in my article.)

1. [Galan JJ](http://www.ncbi.nlm.nih.gov/pubmed?term=Galan JJ%5BAuthor%5D&cauthor=true&cauthor_uid=17099213), [Guarducci E](http://www.ncbi.nlm.nih.gov/pubmed?term=Guarducci E%5BAuthor%5D&cauthor=true&cauthor_uid=17099213), [Nuti F](http://www.ncbi.nlm.nih.gov/pubmed?term=Nuti F%5BAuthor%5D&cauthor=true&cauthor_uid=17099213), [Gonzalez A](http://www.ncbi.nlm.nih.gov/pubmed?term=Gonzalez A%5BAuthor%5D&cauthor=true&cauthor_uid=17099213), [Ruiz M](http://www.ncbi.nlm.nih.gov/pubmed?term=Ruiz M%5BAuthor%5D&cauthor=true&cauthor_uid=17099213), [Ruiz A](http://www.ncbi.nlm.nih.gov/pubmed?term=Ruiz A%5BAuthor%5D&cauthor=true&cauthor_uid=17099213), [Krausz C](http://www.ncbi.nlm.nih.gov/pubmed?term=Krausz C%5BAuthor%5D&cauthor=true&cauthor_uid=17099213): **Molecular analysis of estrogen receptor alpha gene AGATA haplotype and SNP12 in European populations: potential protective effect for cryptorchidism and lack of association with male infertility.** *Hum Reprod* 2007, **22**:444-449.

(The SNP in this article is a different one from the four in my article.)

1. [Feng Z](http://www.ncbi.nlm.nih.gov/pubmed?term=Feng Z%5BAuthor%5D&cauthor=true&cauthor_uid=21402718), [Zhang C](http://www.ncbi.nlm.nih.gov/pubmed?term=Zhang C%5BAuthor%5D&cauthor=true&cauthor_uid=21402718), [Kang HJ](http://www.ncbi.nlm.nih.gov/pubmed?term=Kang HJ%5BAuthor%5D&cauthor=true&cauthor_uid=21402718), [Sun Y](http://www.ncbi.nlm.nih.gov/pubmed?term=Sun Y%5BAuthor%5D&cauthor=true&cauthor_uid=21402718), [Wang H](http://www.ncbi.nlm.nih.gov/pubmed?term=Wang H%5BAuthor%5D&cauthor=true&cauthor_uid=21402718), [Naqvi A](http://www.ncbi.nlm.nih.gov/pubmed?term=Naqvi A%5BAuthor%5D&cauthor=true&cauthor_uid=21402718), [Frank AK](http://www.ncbi.nlm.nih.gov/pubmed?term=Frank AK%5BAuthor%5D&cauthor=true&cauthor_uid=21402718), [Rosenwaks Z](http://www.ncbi.nlm.nih.gov/pubmed?term=Rosenwaks Z%5BAuthor%5D&cauthor=true&cauthor_uid=21402718), [Murphy ME](http://www.ncbi.nlm.nih.gov/pubmed?term=Murphy ME%5BAuthor%5D&cauthor=true&cauthor_uid=21402718), [Levine AJ](http://www.ncbi.nlm.nih.gov/pubmed?term=Levine AJ%5BAuthor%5D&cauthor=true&cauthor_uid=21402718), [Hu W](http://www.ncbi.nlm.nih.gov/pubmed?term=Hu W%5BAuthor%5D&cauthor=true&cauthor_uid=21402718): **Regulation of female reproduction by p53 and its family members.** *FASEB J* 2011, **25**:2245-2255.

(This article is not about male infertility.)

1. [Guarducci E](http://www.ncbi.nlm.nih.gov/pubmed?term=Guarducci E%5BAuthor%5D&cauthor=true&cauthor_uid=16396937), [Nuti F](http://www.ncbi.nlm.nih.gov/pubmed?term=Nuti F%5BAuthor%5D&cauthor=true&cauthor_uid=16396937), [Becherini L](http://www.ncbi.nlm.nih.gov/pubmed?term=Becherini L%5BAuthor%5D&cauthor=true&cauthor_uid=16396937), [Rotondi M](http://www.ncbi.nlm.nih.gov/pubmed?term=Rotondi M%5BAuthor%5D&cauthor=true&cauthor_uid=16396937), [Balercia G](http://www.ncbi.nlm.nih.gov/pubmed?term=Balercia G%5BAuthor%5D&cauthor=true&cauthor_uid=16396937), [Forti G](http://www.ncbi.nlm.nih.gov/pubmed?term=Forti G%5BAuthor%5D&cauthor=true&cauthor_uid=16396937), [Krausz C](http://www.ncbi.nlm.nih.gov/pubmed?term=Krausz C%5BAuthor%5D&cauthor=true&cauthor_uid=16396937): **Estrogen receptor alpha promoter polymorphism: stronger estrogen action is coupled with lower sperm count.** *Hum Reprod* 2006, **21**:994-1001.

(This article is about (TA)_n_ repeats.)

1. [Galan JJ](http://www.ncbi.nlm.nih.gov/pubmed?term=Galan JJ%5BAuthor%5D&cauthor=true&cauthor_uid=16213843), [Buch B](http://www.ncbi.nlm.nih.gov/pubmed?term=Buch B%5BAuthor%5D&cauthor=true&cauthor_uid=16213843), [Cruz N](http://www.ncbi.nlm.nih.gov/pubmed?term=Cruz N%5BAuthor%5D&cauthor=true&cauthor_uid=16213843), [Segura A](http://www.ncbi.nlm.nih.gov/pubmed?term=Segura A%5BAuthor%5D&cauthor=true&cauthor_uid=16213843), [Moron FJ](http://www.ncbi.nlm.nih.gov/pubmed?term=Moron FJ%5BAuthor%5D&cauthor=true&cauthor_uid=16213843), [Bassas L](http://www.ncbi.nlm.nih.gov/pubmed?term=Bassas L%5BAuthor%5D&cauthor=true&cauthor_uid=16213843), [Martinez-Pineiro L](http://www.ncbi.nlm.nih.gov/pubmed?term=Martinez-Pineiro L%5BAuthor%5D&cauthor=true&cauthor_uid=16213843), [Real LM](http://www.ncbi.nlm.nih.gov/pubmed?term=Real LM%5BAuthor%5D&cauthor=true&cauthor_uid=16213843), [Ruiz A](http://www.ncbi.nlm.nih.gov/pubmed?term=Ruiz A%5BAuthor%5D&cauthor=true&cauthor_uid=16213843): **Multilocus analyses of estrogen-related genes reveal involvement of the ESR1 gene in male infertility and the polygenic nature of the pathology.** *Fertil Steril* 2005, **84**:910-918.

(The SNP in this article is a different one from the four in my article.)

1. [Suzuki Y](http://www.ncbi.nlm.nih.gov/pubmed?term=Suzuki Y%5BAuthor%5D&cauthor=true&cauthor_uid=12477541), [Sasagawa I](http://www.ncbi.nlm.nih.gov/pubmed?term=Sasagawa I%5BAuthor%5D&cauthor=true&cauthor_uid=12477541), [Itoh K](http://www.ncbi.nlm.nih.gov/pubmed?term=Itoh K%5BAuthor%5D&cauthor=true&cauthor_uid=12477541), [Ashida J](http://www.ncbi.nlm.nih.gov/pubmed?term=Ashida J%5BAuthor%5D&cauthor=true&cauthor_uid=12477541), [Muroya K](http://www.ncbi.nlm.nih.gov/pubmed?term=Muroya K%5BAuthor%5D&cauthor=true&cauthor_uid=12477541), [Ogata T](http://www.ncbi.nlm.nih.gov/pubmed?term=Ogata T%5BAuthor%5D&cauthor=true&cauthor_uid=12477541): **Estrogen receptor alpha gene polymorphism is associated with idiopathic azoospermia.** *Fertil Steril* 2002, **78**:1341-1343.

(The SNP in this article is a different one from the four in my article.)

1. [Heimdal K](http://www.ncbi.nlm.nih.gov/pubmed?term=Heimdal K%5BAuthor%5D&cauthor=true&cauthor_uid=7742719), [Andersen TI](http://www.ncbi.nlm.nih.gov/pubmed?term=Andersen TI%5BAuthor%5D&cauthor=true&cauthor_uid=7742719), [Skrede M](http://www.ncbi.nlm.nih.gov/pubmed?term=Skrede M%5BAuthor%5D&cauthor=true&cauthor_uid=7742719), [Fosså SD](http://www.ncbi.nlm.nih.gov/pubmed?term=Fosså SD%5BAuthor%5D&cauthor=true&cauthor_uid=7742719), [Berg K](http://www.ncbi.nlm.nih.gov/pubmed?term=Berg K%5BAuthor%5D&cauthor=true&cauthor_uid=7742719), [Børresen AL](http://www.ncbi.nlm.nih.gov/pubmed?term=Børresen AL%5BAuthor%5D&cauthor=true&cauthor_uid=7742719): **Association studies of estrogen receptor polymorphisms in a Norwegian testicular cancer population.** *Cancer Epidemiol Biomarkers Prev* 1995, **4**:123-126.

(This article is not about male infertility.)
